# Virtual Screening as a Technique for PPAR Modulator Discovery

**DOI:** 10.1155/2010/861238

**Published:** 2009-09-02

**Authors:** Stephanie N. Lewis, Josep Bassaganya-Riera, David R. Bevan

**Affiliations:** ^1^Genetics, Bioinformatics, and Computational Biology Program, Virginia Polytechnic Institute and State University, Blacksburg, VA 24061, USA; ^2^Department of Biochemistry, Virginia Polytechnic Institute and State University, 201 Engel Hall 0308, Blacksburg, VA 24061, USA; ^3^Nutritional Immunology and Molecular Nutrition, Virginia Bioinformatics Institute, Virginia Polytechnic Institute and State University, Washington Street 0477, Blacksburg, VA 24061, USA

## Abstract

Virtual screening (VS) is a discovery technique to identify novel compounds with therapeutic and preventive efficacy against disease. Our current focus is on the in silico screening and discovery of novel peroxisome proliferator-activated receptor-gamma (PPAR*γ*) agonists. It is well recognized that PPAR*γ* agonists have therapeutic applications as insulin sensitizers in type 2 diabetes or as anti-inflammatories. VS is a cost- and time-effective means for identifying small molecules that have therapeutic potential. Our long-term goal is to devise computational approaches for testing the PPAR*γ*-binding activity of extensive naturally occurring compound libraries prior to testing agonist activity using ligand-binding and reporter assays. This review summarizes the high potential for obtaining further fundamental understanding of PPAR*γ* biology and development of novel therapies for treating chronic inflammatory diseases through evolution and implementation of computational screening processes for immunotherapeutics in conjunction with experimental methods for calibration and validation of results.

## 1. Introduction

Transdisciplinary research has become a common means of addressing the most pressing societal problems. Past discoveries of scientific hallmarks have favored exploring the depths of established ideas across scientific disciplines to better understand biological systems and processes. This is possible because the wealth of scientific knowledge has only scratched the surface of how biological systems work, and often exploring the unknown intricacies of biological networks requires knowledge of more than one scientific realm. 

 The extreme amounts of information readily available to the scientific community present a valuable and perpetually renewing resource. However, this overabundance also poses a problem. There is simply too much information within too many areas of science for one person with expertise in a single field to rapidly make novel advances. Take, for example, the question of what factors determine whether an individual suffers from a particular disease. When designing a treatment, one can look at the symptoms, the cause of the symptoms, genetic differences between healthy and afflicted individuals, genetic differences between individuals with the same disease but slightly different symptoms, methods for treating the symptoms, methods for controlling or correcting the disease, and methods for screening for the disease. This list includes, but is not limited to, disciplines such as genetics, bioinformatics, biochemistry, pharmacology, and medicine, and it is the combination of all these disciplines that facilitates the development of effective preventive and therapeutic approaches. 

 In a more general sense, there is also an increasing need for integrating computational and experimental approaches. Computers have become a large and vital part of scientific exploration and serve to simplify and expedite processes that could take months to years for an individual to complete. First, computers allow for organization of scientific knowledge. Second, they allow for sharing of ideas and discoveries in an effective and timely fashion. Third, computers allow individuals to better analyze experimental results and develop more efficacious test methods. The fourth and ultimate benefit of computer technology to science is improved efficiency due to a reduced necessity for time, money, and resources. 

 Peroxisome proliferator-activated receptor (PPAR) research is one of many areas that may benefit from advances in computational biology and other transdisciplinary approaches. Mixtures of computational and experimental studies have given insight into characteristics of PPARs, particularly PPAR-gamma (PPAR*γ*) and its modulators, as well as the role of these proteins in treating type 2 diabetes (T2D), gastrointestinal diseases, and genetic disorders associated with glucose homeostasis and lipid uptake.

## 2. Characteristics of PPAR*γ* and the Activation Process

PPAR*γ* is one of three known PPAR isoforms (*α*, *δ*, and *γ*). PPARs belong to the nuclear hormone receptor superfamily and have been found to regulate inflammation, immunity, and metabolism [1, 2]. Members of this superfamily are structurally and functionally conserved transcription factors that regulate both target gene expression and repression after ligand binding occurs [3]. A diverse set of natural and synthetic molecules is classified as ligands that can induce activation and expression of PPARs. These ligands include nutrients, nonnutrient endogenous ligands, and drugs such as thiazolidinediones (TZDs) and fibrates [1, 2, 4]. Known endogenous and dietary agonists include conjugated linoleic acid (CLA), 9-(S)-hydroxyoctadecadienoic (9-HODE), 13-(S)-hydroxyoctadecadienoic (13-HODE) acid, and 15-deoxy-Δ^12,14^-prostaglandin J2 (15d-PGJ2) [1, 5]. 

 A great deal of literature focuses on increasing insulin sensitivity by controlling PPAR*γ* interactions and altering gene expression of various transcription factors. PPAR*γ* is a component of an extensive group of controls for adipogenesis and glucose homeostasis, and both of these processes directly affect obesity and T2D [6]. PPAR*γ* is located in high concentrations in adipocytes, and has also been found in significant amounts in the retina, cells of the immune system, and colonic epithelial cells [1, 7]. Functionally, PPAR*γ* downregulates the expression of proinflammatory cytokines by antagonizing the activities of transcription factors such as AP-1 and NF-*κ*B, and favoring the nucleocytoplasmic shuttling of the activated p65 subunit of NF-*κ*B [2]. As a consequence of the important roles PPARs play in controlling metabolic homeostasis and inflammatory processes, they are all well recognized as molecular targets for drugs against metabolic diseases, such as T2D [8–10], and treatment of immunoinflammatory disorders. 

 Structurally, PPAR*γ* is composed of a DNA-binding domain (DBD), a hinge region, and a ligand-binding domain (LBD). The first step in PPAR*γ* activation is disassociation of corepressors after binding of retinoic acid (RA) to a single retinoid X receptor (RXR) subunit. This step is an essential part of numerous endocrine system pathways [6]. The ligand-bound RXR then associates with ligand-bound PPAR*γ*. To become fully active, the PPAR*γ*-RXR*α* heterodimer requires association of coactivator molecules [6]. Agonist binding to PPAR*γ* regulates activity by causing conformational changes to the LBD, which is composed of approximately 250 amino acids near the C-terminal end of the protein [11]. Mediation of activity is a direct result of changes to the transcription activation function-2 (AF-2) domain [6, 12]. These changes vary depending on the type of ligand that binds to the LBD. Changes to AF-2 allow for coactivator recruitment, followed by transcriptional activation. 

 Co-activator recruitment is based on a LXXLL binding motif (nuclear receptor box) found on both PPAR*γ* and coactivators like steroid receptor coactivating factor-1 (SRC-1) that associate for transcription induction after the conformational change of the AF-2 region [3, 13, 14]. The DNA binding domains of PPAR*γ*-RXR*α* interact with PPAR response elements (PPREs) found within the genome [15]. Such elements include 5′ regions for aP2 and PEPCK genes as part of adipogenesis, which suggests PPAR*γ* plays a major role in fat cell-specific gene function [15]. Though PPAR*γ* is typically known to interact with DNA, it can also interact directly with other proteins to induce activity. For example, as preadipocytes differentiate, expression of C/EBP*β* and C/EBP*δ* directly activate PPAR*γ* and C/EBP*α*, which promote further differentiation and full insulin sensitivity [15]. Alternatively, binding by specific ligands can induce activity as well. The use of TZDs in the treatment of T2D improves insulin resistance by increasing GLUT-4 levels and decreasing the levels of cytokines that induce insulin resistance, such as TNF-*α* and IL-6 [15] by antagonizing the activity of proinflammatory transcription factors [2]. Therefore, it is important to note that understanding the interactions involved in coactivator recruitment is crucial for predicting activity after ligand binding, and ultimately treatment of insulin insensitivity and inflammation.

## 3. Agonists and the Ligand-Binding Domain of PPAR*γ*

Fatty acids and lipid metabolites have been found to be endogenous ligands for PPAR*γ*. A recent study by Waku et al. [16] gives insight into how these ligands bind covalently to Cys285, thereby modifying PPAR*γ* conformations. In particular, these covalent modifications induce rearrangement of the side-chain network around the created covalent bond in order to generate different transcriptional strengths. This attenuation of strength is specific to the ligand type and conformation. Waku et al. also mention that Ile267 and Phe287 are two key residues repositioned by covalent binding of fatty acids [16]. It is also important to note for some fatty acids, formation of a complex containing two fatty acid units is necessary for binding within the LBD of PPAR*γ* [5]. 

 Synthetic ligands that can interact with PPAR*γ* can be divided into at least three classes: full agonists, partial agonists, and antagonists. Full agonists bind and alter the conformation of the AF-2 domain allowing for coactivators to bind for activation of genes for both adipogenic and insulin sensitivity processes. Partial agonist binding leads to a change that allows for recruitment of coactivators responsible for insulin sensitivity without affecting adipogenesis. Antagonists show high affinity, but do not activate PPAR*γ*, suggesting the conformational change to AF-2 is either not enough to allow coactivator association or is similar to that of the inactive conformation. A study conducted by Kallenberger et al. showed that the dynamics of the AF-2 region plays a major role in the genetic regulation capabilities of PPAR*γ*. Binding of a ligand reduces AF-2 mobility and allows for regulation of gene expression. Furthermore, the AF-2 region of PPAR*γ* can undergo natural mutations, which result in severe insulin insensitivity and cause noticeable changes in dynamics of that AF-2 region [12]. 

**Figure 1:**
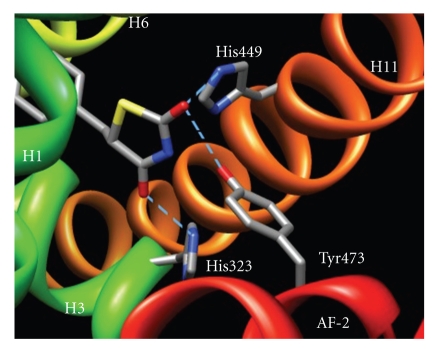
Rosiglitazone bound to LBD of PPAR*γ*. Helices are labeled with H, followed by a number. Key residues involved in hydrogen bonding are labeled. Blue dashed lines represent bonding interactions between the hydrogens of the residue and the oxygens of the ligand. (PDB ID 3DZY).

**Figure 2:**
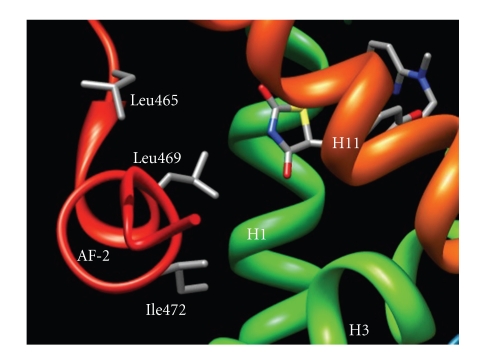
Rosiglitazone bound to LBD of PPAR*γ*. Helices are labeled with H, followed by a number. Some of the key residues involved in hydrophobic interactions are labeled. (PDB ID 3DZY).

 PPAR*γ* agonists typically possess a small polar region and a hydrophobic region that form hydrogen bonds and hydrophobic interactions, respectively, within the LBD. Hydrogen bonding typically occurs between His323, Tyr473, and His449 of the PPAR*γ* LBD and carbonyl oxygens of the ligand (Figure 1) [6, 13, 17]. Hydrogen bonding of the ligand to Tyr473 is key to the stabilization of the AF-2 region [13, 18]. The hydrophobic moiety interacts with other residues in the cavity, such as Leu465, Leu469, and Ile472, establishing hydrophobic interactions to stabilize the domain (Figure 2) [6, 13, 17]. 

 In the case of partial agonists, key interactions are different, which result in lesser degrees of AF-2 stabilization and differential stabilization of distinct regions of the LBD [5]. Either of these events leads to activation as a result of a shift of the ligand polar group away from the hydrogen-bonding residues. This shift can prevent hydrogen bonding or lead to a different hydrogen-bonding network. Changes in the hydrophobic interactions between the ligand and residues within the LBD also exist. The combination of these events results in conformational changes different enough from those caused by full agonist binding to elicit only partial activation and recruitment of different coactivators [5, 17]. 

 Antagonists for PPAR*γ* have not received the same amount of research interest as the full and partial agonists. Therefore, little information is available on the binding of this type of ligand to the *γ* isoform. Antagonists for PPAR*α*, however, have provided insight into how ligands of this class might interact with PPAR*γ* due to the conservation of the mode of corepressor binding. Typically, corepressors bind to PPAR*α* in the absence of ligand. The complex is then stabilized by antagonists, which disrupt any potential interactions with coactivators, and thereby prevent the initiation of transcription [19]. 

 The LBD of PPAR*γ* is a large, T-shaped cavity [17] with a volume of approximately 1440 Å^3^ [6, 17], which can easily accommodate many different ligands due to the dynamics of the ligand-binding pocket [20]. It is important to note that the type of ligand determines which coactivator associates with the PPAR*γ*-RXR*α* heterodimer. The coactivator then determines the target gene for regulation and the direction of regulation (up or down). Thus, knowing the final conformation of the LBD that is necessary to elicit a specific activity is crucial for therapeutic development [3]. 

 Until recently, available crystal structures for PPAR*γ* generally were composed solely of the PPAR*γ* LBD with a ligand bound, a RXR*α* LBD heterodimerized to PPAR*γ*, and a short segment of a coactivator protein. Chandra et al. have published three new crystal structures (3DZU, 3DZY, and 3E00) for PPAR*γ* composed of the DBD, the hinge region, and the LBD with ligand bound [21]. These structures are in complex with RXR*α*, polypeptides that mimic the LXXLL motif for coactivator binding, and a short DNA segment representative of a PPRE. Observations related to heterodimerization of PPAR*γ* and RXR*α*, as well as activation of response elements are reported in this study. The LBD and DBD of PPAR*γ* are positioned closely together, which aids in coupling of the PPAR*γ* LBD to the relatively wide space between the LBD and DBD of RXR*α* [21]. The study also discusses the polarity of the PPAR*γ*-RXR*α* heterodimer, which is determined by the (C)-terminal extension of PPAR*γ* and the DBD interactions of the two subunits. Table 1 contains a list of all currently available structures for PPAR*γ*, which can be found in RCSB's PDB online database [22] http://www.pdb.org/.

## 4. Docking

Docking can be defined as predicting both ligand conformation and orientation within a targeted binding site [52]. Experimentally derived crystal and NMR protein structures are used as the basis for docking, and the physics involved is based on what is known about atomic and molecular interactions, as well as laws of thermodynamics. All docking methods must include sampling ligand conformations, generating poses of the ligand within the receptor binding site, and scoring the poses. 

 Before beginning a docking study, one must select from three conformational searching methods: systematic, random, and simulation. The systematic method explores the degrees of freedom possessed by the torsional bonds of a molecule. To achieve this goal, the ligand parts are introduced incrementally in order to obtain an energetically favorable conformation. Random searching, as the name implies, is based on generating random torsional variations of an initial conformation to test against the target. Simulation methods utilize molecular dynamics and energy minimization, and serve best when coupled with one or both of the above searching methods [52]. 

A large number of docking and dynamics software packages and online servers exist (Table 2), many of which are freely available for academic research. The variations in calculation methods and results make each program slightly different. Therefore, the researcher must pick which docking programs are ideal for his or her study. Studies have been performed to assess which programs are ideal for specific screening approaches or particular protein families. For instance, Kellenberger et al. published a comparative evaluation of eight widely used docking programs for screening accuracy in 2004 [53]. Of the eight docking programs tested, GLIDE, GOLD, and SURFLEX provided the best docking and ranking accuracy within a 2.0 Å cutoff for root-mean squared deviation (RMSD), whereas QXP showed promising docking accuracy but reduced ranking performance. For ranking, FlexX outperformed QXP with percent scoring errors of 15% and 55%, respectively. Efficacy in screening of a compound database was found with SURFLEX, with 8 hits for ligands that bind to a difficult target out of 50 total compounds. GLIDE, GOLD, and FlexX were deemed good programs for virtual screening with hit values of 5, 4, and 4, respectively. Regarding docking times, FRED, which did not perform as well with scoring and docking accuracy, took the least amount of time to perform docking calculations of the eight programs tested, followed by DOCK and FlexX. No single program was deemed the best docking software, but the study demonstrated that the characteristics of the ligand and the target have a significant effect on the efficiency of the docking program used [53].

## 5. Virtual Screening

Because the process of finding a novel compound showing bioactivity can be time-consuming and expensive, structure-based drug design has been established as a vital first step to therapeutic development [54]. Screening for ligand conformations can be performed using a ligand-based or a structure-based approach [55, 56]. Ligand-based design uses known active and inactive compounds to generate a pharmacophore [55], which is often used in conjunction with quantitative structure-activity relationship (QSAR) analysis to determine ligand-protein interactions. Receptor-based design requires the availability of the receptor structure, which is used to examine the interactions that occur with any members of a large database of ligands [56]. Computational screening of large databases of molecules against the three-dimensional structure of a protein has the potential to provide rapid and accurate prediction of the binding modes and affinities of possible hits for lead optimization. One can prescreen a database of thousands of compounds and narrow the field of ligands to two or three potential hits in a significantly reduced amount of time compared to laboratory experimental methods. This smaller group would increase the efficiency of experimental assays and new agonist discovery. Virtual Screening, which incorporates high-throughput docking techniques, is a means to explore the LBD of a protein and make predictions about ligand binding. This technique categorizes ligands that bind to the protein of interest and allows predictions to be made about activation or inhibition of the protein. 

 Development begins with creating an algorithm that can be followed to set up the testing, run the testing, and finally analyze the results. Schneider and Böhm define these three issues that must be addressed when performing iterative structure generation respectively as, the construction problem, the docking problem, and the scoring problem [57]. Deciding which protein crystal structure to use for all ligands, establishing a set of test parameters, and deciding which ligands to include in the test library make up the first part of the process. Typically, a crystal structure with the highest resolution and fewest missing atoms and residues will be selected. Setting parameters involves re-docking of published structures to reproduce experimentally observed docking conformations [52]. The compound database, which can contain numbers of compounds in the thousands [58], should contain small molecules that, based on known chemical interactions between residues of the binding site and known ligands, will bind in varying degrees to the protein of interest and potentially yield the desired effect (e.g., conformational change and activation or inhibition of protein). Protein flexibility is also an important and necessary part of predicting orientations and interactions for many protein families [54], and therefore time should be taken to consider how to incorporate receptor flexibility as well as the binding site microenvironment (i.e., water and/or ions in the binding site). Once the conditions for docking have been established, docking, which is the second step, is relatively straightforward. 

 The final step, analysis, can often be the most daunting due to the variety of ways output can be interpreted and analyzed. The type of program used to perform simulations has a significant effect on analysis methods because of the information returned. Some programs may be better suited for calculating inhibition constants and free energy of binding estimations than others, whereas still other programs may provide more variables for consideration. There are many different approaches to analyzing results that one can take for scoring the results of a docking study, and these approaches involve examination of interactions on either a fragment or atomic level [57].

## 6. Limitations of Virtual Screening

Because VS is not, as of yet, a stand-alone process, ligand-binding and reporter assays are essential processes for validating in silico results. Docking predicts ligands that may elicit the desired activity, and assays further refine the group of viable candidates to a select group of hits, which, at a specific concentration, will activate the protein of interest. 

 Further research into lesser-understood biochemical processes is necessary to improve upon the reliability of VS as a stand-alone process. These processes include protein flexibility and induced-fit adaptations, the role of water in solvation, desolvation and ligand binding, and the involvement of electrostatics [52, 59]. Though these unknowns can prove to be problematic when looking at a single computational method, combining strategies is a way to improve upon successful hit rates. Overall, VS saves time and resources when searching libraries of compounds to narrow candidates down to a handful of potential hits that can then be tested experimentally. There is also potential to find hits that may not have been discovered using experimental processes alone. 

 Another factor that can limit VS productivity is the amount of information available when building a compound library. An information-rich environment is available when considering natural compounds for the treatment and prevention of diseases. Natural plant extracts typically contain a vast number of components that one would need to sift through in order to find the one compound or multiple synergistic compounds that elicit a desired mechanistic affect (i.e., activation of PPARs). VS would prove useful after fractionation of natural extracts and chemical elucidation of key peaks to aid in identifying which compounds within a library are the bioactive compounds. This has the potential to minimize the need for serial HTS when testing for a lead candidate. It is important to note that fractionation is not a necessary step for VS, but can be useful for guiding database building when examining natural extracts for bioactivity.

## 7. Docking and Virtual Screening Successes

Despite the present inability of VS to replace HTS, the two can be complementary approaches to candidate pharmaceutical and nutraceutical searching because of the potential for one method to find activators or inhibitors for which the other method does not show results [59]. Klebe [59] mentions in a review of VS strategies a comparison study performed by two groups searching for *Escherichia coli* dihydrofolate reductase inhibitors from a database of approximately 50,000 compounds. The VS portion of the study revealed a number of compounds previously unknown as inhibitors due to insufficient concentrations of the compounds being used during experimental testing [59–62]. Klebe also provides a list of targets that have previously been addressed by virtual screening. These targets include nuclear receptors such as retinoic acid receptor and thyroid hormone receptor [59].

## 8. Relevance to PPAR*γ* Agonist Discovery

Docking techniques would prove useful in the development of new PPAR-based therapeutics, including in silico screening of synthetic agonists and natural compounds from plant extracts (i.e., botanicals), all of which have shown promise in the treatment and prevention of immunoinflammatory diseases through PPAR*γ* agonism. Docking and simulation techniques provide a means to prescreen for and enrich compounds with PPAR*γ* agonism and thereby increase the efficiency of HTS. Docking also allows for structure-based searches for analogues and derivatives of known agonists. 

 To date, there have been several studies utilizing standard docking methods [63–66] and VS methods [60, 63, 67–70]. Most of these studies focus on derivatives or analogues of a particular compound showing high affinity for PPAR*γ*. Studies of this nature can serve two purposes: identify hits for therapeutic development and provide insight into ligand-protein interactions and ligand selectivity. 

 Xu et al. [64] published a study in 2003 in which docking methods were used to look at interactions between PPAR*γ* and eighteen known synthetic and natural agonists in order to determine the pharmacophore of PPAR*γ* agonists. The group determined that PPAR*γ* agonists must have a polar head group and hydrophobic tail in order to form necessary hydrogen bonding and hydrophobic contacts with the LBD, respectively [64]. 

 In another study, Lu et al. [63] conducted a structure-based VS search for PPAR*γ* partial agonists as candidates for treatment of T2D with fewer side effects than full agonists. The search revealed a class of ligands that could then be used to test against PPAR*γ*. Two compounds of the class were identified as partial agonists with selectivity among the three PPAR subtypes, and would serve as candidates for further testing. Using VS, they were able to suggest determinants in ligand specificity. The computational results were coupled with X-ray crystallography and assessment of in vitro and in vivo protein activity [63]. 

 A study regarding natural products identified as PPAR*γ* agonists conducted by Salam et al. [68] also utilized structure-based VS to identify 29 potential agonists for experimental testing. Of those compounds, 6 were found to induce PPAR*γ* transcriptional activity in vitro. The study also provided insight into the mechanism underlying the flavonoid-induced conformational change and activation of PPAR*γ* [68].

## 9. Future Directions

Naturally occurring compounds with preventive or therapeutic activity (nutraceuticals) represent a widely used Complementary and Alternative Medicine (CAM) modality and are an alternative to pharmaceuticals (i.e., TZDs) for treating various chronic diseases such as T2D. These natural compounds can modulate gene expression [71] and are typically safer than synthetic counterparts. In the case of T2D, nutraceuticals have the potential to decrease the risk of myocardial infarction, weight gain, and edema associated with current synthetic PPAR*γ* agonist treatments [10, 72]. Unfortunately, finding a compound that elicits a desired activity is not always easy because isolating a single compound from a bioactive extract is time consuming and expensive [73] and the mechanism by which the compound works is often unknown [74]. VS, in combination with conventional experimental methods, has the potential to put the discovery of bioactive botanical constituents in a better competitive position with mainstream pharmaceutical research by reducing time and costs. For instance, in a study published by Rollinger et al. [75], a chemical feature-based pharmacophore modeling VS technique, in combination with ethnopharmacology, was utilized to identify inhibitors for cyclooxygenase (COX) I and II. Of the thousands of compounds listed in the four databases used (WDI, NCI, NPD, and DIOS), the success rate of finding known inhibitors within these three-dimensional databases was enhanced through the use of VS techniques [75]. 

 A preliminary comparison of a small group of PDB PPAR*γ* structures shows an overall conservation of backbone conformation across the available structures. This is relevant to the selection of a single macromolecule for large-scale automated testing. These findings suggest it is possible to select one macromolecule for all ligand types with a limited degree of error. It is important to note that though there is a relative consensus position for all key residues, some variation in the positions of key residues due to ligand interactions are present. Therefore, this issue must be considered and several structures must be examined when deciding on a single macromolecule crystal structure for VS. 

Another computational method that may prove useful is molecular dynamics (MD), which involves the use of computational chemistry to predict the dynamics of complex molecular systems and the macroscopic properties of those systems based on detailed atomic knowledge [76]. Implementing MD would prove useful for examination of conformational changes and molecular interactions, which would allow for expansion upon what is known about how PPARs interact with ligands and other macromolecules. 

To discover potential nutraceutical/CAM hits, further assessment of PPAR*γ* and ligand characteristics is necessary to determine the best screening approach and which scoring functions compare for analysis. If the components of an extract are known or if one can speculate as to which compounds are present, a database of chemically related compounds could be created to test against PPAR*γ*, and a smaller hit group can be identified for experimentation. Another necessary element is collecting experimentally proven properties for comparison to computationally derived data. Future work could also encompass finding coagonists and pan-agonists for PPAR subtypes.

## Figures and Tables

**Table 1:** *PDB IDs of published crystal and NMR structures for PPARγ* *with various ligands bound*. Resolution values are in Angstroms (Å). The “Reference *number*” columns list references for each PDB ID. The citations for these are present in the reference section. All PDB IDs list *Homo sapiens* as the protein source except 1ZGY (*Rattus norvegicus*). Access date: 23 March 2009.

Structure ID	Resolution (Å)	Release date	Reference number	Structure ID	Resolution (Å)	Release date	Reference number
1FM6	2.1	02/16/01	[6]	2Q6S	2.4	10/23/07	[23]
1FM9	2.1	02/16/01	[6]	2Q8S	2.3	10/14/08	[24]
1I7I	2.3	03/09/02	[25]	2QMV	n/a (NMR)	09/02/08	[26]
1K74	2.3	12/05/01	[27]	2VSR	2.0	08/19/08	[5]
1KNU	2.5	12/19/02	[28]	2VST	2.3	08/19/08	[5]
1NYX	2.7	07/15/03	[29]	2VV0	2.5	08/19/08	[5]
1PRG	2.2	01/13/01	[13]	2VV1	2.2	08/19/08	[5]
1RDT	2.4	11/09/04	[30]	2VV2	2.8	08/19/08	[5]
1WM0	2.9	09/07/04	[31]	2VV3	2.8	08/19/08	[5]
1ZEO	2.5	04/25/06	[32]	2VV4	2.3	08/19/08	[5]
1ZGY	1.8	07/26/05	[33]	2ZK0	2.4	2/24/09	[16]
2ATH	2.3	08/25/06	[34]	2ZK1	2.6	2/24/09	[16]
2F4B	2.1	02/14/06	[35]	2ZK2	2.3	2/24/09	[16]
2FVJ	2.0	05/16/06	[36]	2ZK3	2.6	2/24/09	[16]
2G0G	2.5	05/16/06	[37]	2ZK4	2.6	2/24/09	[16]
2G0H	2.3	05/16/06	[37]	2ZK5	2.5	2/24/09	[16]
2GTK	2.1	09/26/06	[38]	2ZK6	2.4	2/24/09	[16]
2HFP	2.0	09/19/06	[39]	3B3K	2.6	10/28/08	[40]
2HWQ	2.0	08/07/07	[41]	3BC5	2.3	11/18/08	[42]
2HWR	2.3	08/07/07	[41]	3CDP	2.8	1/13/09	[43]
2I4J	2.1	04/17/07	[17]	3CDS	2.7	12/30/08	[40]
2I4P	2.1	04/17/07	[17]	3CS8	2.3	06/03/08	[44]
2I4Z	2.3	04/17/07	[17]	3CWD	2.4	07/08/08	[45]
2OM9	2.8	04/24/07	[46]	3D24	2.1	06/10/08	[47]
2P4Y	2.3	01/08/08	[48]	3D6D	2.4	12/30/08	[40]
2POB	2.3	03/18/08	[49]	3DZU	3.2	10/28/08	[21]
2PRG	2.3	07/19/99	[13]	3DZY	3.1	10/28/08	[21]
2Q59	2.2	10/23/07	[23]	3E00	3.1	10/28/08	[21]
2Q5P	2.3	10/23/07	[23]	3ET0	2.4	2/17/09	[50]
2Q5S	2.0	10/23/07	[23]	3ET3	2.0	2/17/09	[50]
2Q61	2.2	10/23/07	[23]	3PRG	2.9	08/30/99	[3]
2Q6R	2.4	10/23/07	[23]	4PRG	2.9	05/27/99	[51]

**Table 2:** *List of some commonly used molecular dynamics and docking software packages with developer URL*. This is not intended to be a comprehensive list of all available dynamics and docking programs available. Available programs are typically free to download for academic use, but some require the purchase of a license for use.

Dynamics		Docking	
Program	Developer Site	Program	Developer site
Amber	http://ambermd.org/	AutoDock	http://autodock.scripps.edu/
AMMP	http://www.cs.gsu.edu/~cscrwh/progs/progs.html	FlexX	http://www.biosolveit.de/FlexX/
BALLview	http://www.ballview.org/	FRED	http://www.eyesopen.com/
CHARMM	http://www.charmm.org/	GLIDE	http://www.schrodinger.com/
GROMACS	http://www.gromacs.org/	GOLD	http://www.ccdc.cam.ac.uk/
LOOS Library	http://membrane.urmc.rochester.edu/Software	Sculptor	http://sculptor.biomachina.org/
YASARA	http://www.yasara.org/	SLIDE	http://www.bch.msu.edu/
YUP	http://rumour.biology.gatech.edu/YammpWeb/	SURFLEX	http://www.tripos.com/
ZMM	http://www.zmmsoft.com/	UCSF DOCK	http://dock.compbio.ucsf.edu/
